# Address on Hospital and Dispensary Clinics

**Published:** 1888-07

**Authors:** 


**Address on Hospital and Dispensary Clinics, and the Art of Prescribing.** By Prosper Bender, M. D., Boston.

Both of these pamphlets have been published by Dr. Bender in the *New England Medical Gazette.* The essay upon diabetes has been re-written since its first publication; in it Dr. Bender gives his views upon the physiology and pathology of this disease.

The second is an address, delivered February the 9th, before the Hahnemannis Societas of the Boston U. S. of Me licine. In this address some very sound and practical advice is given upon the art of prescribing, which we would gladly quote in full did our space allow it.

